# Supplementary material for: Progenitor Cells Activated by Platelet Lysate in Human Articular Cartilage as a Tool for Future Cartilage Engineering and Reparative Strategies
Source: Cells. 2020 Apr 23;9(4):1052. doi: 10.3390/cells9041052 (PMC7226425; doi:10.3390/cells9041052)
Supplement: Supplementary file 1 [file cells-09-01052-s001.pdf]

Supplementary Materials

Progenitor Cells Activated by Platelet Lysate in Human Articular Cartilage as a Tool for Future Cartilage Engineering and Reparative Strategies

Simonetta Carluccio [1,†], Daniela Martinelli [1,†], Maria Elisabetta Federica Palamà [1], Rui Cruz Pereira [1,2], Roberto Benelli [3], Ana Guijarro [1], Ranieri Cancedda [4] and Chiara Gentili [1,5,*]

[1] Regenerative Medicine Laboratory, Department of Experimental Medicine (DIMES), University of Genova, via Leon Battista Alberti 2, 16132 Genova, Italy.simonetta.carluccio@edu.unige.it (S.C.); daniela_martinelli7@hotmail.com (D.M.); elisabettapalama@gmail.com (M.E.F.P.); ana.isabel.guijarro.anton@edu.unige.it (A.G.-A.); chiara.gentili@unige.it (C.G)

[2] Neurobiology of miRNA, Fondazione Istituto Italiano di Tecnologia, 16163 Genoa, Italy; rui.pereira@iit.it

[3] UOSD Oncologia Molecolare e Angiogenesi, IRCCS Ospedale Policlinico San Martino, largo Rosanna Benzi 10, 16132 Genoa, Italy; roberto.benelli@hsanmartino.it

[4] Endolife S.r.l., Piazza della Vittoria 15/23, 16121 Genova, Italy; ranieri.cancedda@unige.it

[5] Center for Biomedical Research (CEBR), University of Genova, viale Benedetto XV 9, 16132 Genova, Italy. chiara.gentili@unige.it (C.G)

* Correspondence: chiara.gentili@unige.it

† These authors contributed equally to this work.

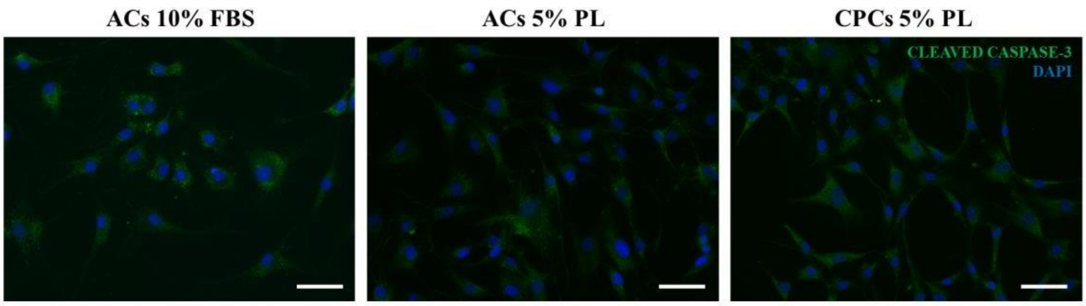

Figure S1. Immunofluorescence staining for cleaved-caspase 3 in ACs-FBS, ACs-PL and CPCs-PL. All scale bars correspond to 100 μm.

Table S1. In vitro analysis of ACs and CPCs secretory profiles. Quantification of the mean pixel density for each identified cytokine (analyte) in the three experimental groups (ACs-FBS, ACs-PL and CPCs-PL). Data were represented as mean and uncertainty in measurement for each spot couple in the array membranes.

Analyte	ACs 10% FBS			ACs 5% PL			CPCs 5% PL		
ApoA1	4050	±	300	35950	±	140	32110	±	820
Angiogenin	63830	±	200	60010	±	2800	61420	±	400
BAFF	31440	±	250	6610	±	160	14850	±	2780
CD14	11960	±	1470	24200	±	1380	3950	±	320
CHI3L1	62230	±	960	33390	±	170	50280	±	190
CST3	17320	±	2620	15700	±	1530	29710	±	160
Dkk-1	660	±	100	3940	±	470	35070	±	160
DPPIV	240	±	60	180	±	80	31030	±	1410
CD147	28060	±	80	42260	±	1390	38410	±	840
CXCL5	420	±	140	61640	±	130	32760	±	930
CD105	38630	±	100	32210	±	830	21220	±	250
GDF-15	45850	±	660	54810	±	270	38110	±	2610
CXCL1	2670	±	520	51050	±	1000	494520	±	1180
IGFBP-2	61570	±	560	51520	±	540	8780	±	90
IGFBP-3	48000	±	700	47660	±	960	52660	±	170
IL-6	17780	±	310	58410	±	100	8860	±	1010
IL-8	44810	±	540	60480	±	220	57810	±	490
LIF	3130	±	20	25830	±	1180	7610	±	200
NGAL	11630	±	250	38030	±	70	9190	±	340
CCL2	53880	±	960	55070	±	360	58590	±	680
CCL7	41990	±	320	49180	±	540	53730	±	340
MIF	30690	±	250	33900	±	40	38280	±	1370
CCL20	1140	±	60	61020	±	1690	1270	±	120
MMP9	2340	±	120	47430	±	340	14830	±	1990
OPN	58280	±	570	54770	±	1080	7360	±	250
PTX3	51560	±	910	53010	±	380	58290	±	170
CXCL4	220	±	120	42060	±	120	2060	±	1340
CCL5	430	±	40	43340	±	560	45400	±	330
RBP-4	40060	±	760	46900	±	540	2570	±	450
CXCL12	2000	±	400	18470	±	850	37250	±	1630
Serpin E1	55880	±	280	29490	±	310	30500	±	200
ST2	41350	±	600	36380	±	830	1520	±	290
THBS1	43860	±	490	40080	±	720	27430	±	1000
uPAR	27150	±	1080	49840	±	120	42230	±	810
VEGF	48520	±	100	56910	±	650	60110	±	740
VDB	5630	±	700	32990	±	1200	18530	±	630
CD106	58460	±	10	54230	±	580	28480	±	190
